# Very low expression of PD-L1 in medullary thyroid carcinoma

**DOI:** 10.1530/ERC-17-0104

**Published:** 2017-04-18

**Authors:** Massimo Bongiovanni, Caterina Rebecchini, Chiara Saglietti, Jean-Luc Bulliard, Laura Marino, Laurence de Leval, Gerasimos P Sykiotis

**Affiliations:** 1 Service of Clinical PathologyLausanne University Hospital, Institute of Pathology, Lausanne, Switzerland; 2 Institute of Social and Preventive MedicineLausanne University Hospital, Lausanne, Switzerland; 3 Service of EndocrinologyDiabetology and Metabolism, Lausanne University Hospital, Lausanne, Switzerland

## Dear Editor,

Immunotherapy inhibiting the signaling interaction between programmed death 1 (PD1) and its ligand programmed death-ligand 1 (PD-L1) is rapidly expanding as an established or experimental oncological treatment for several types of solid tumors, especially melanoma and non-small cell lung carcinoma (NSCLC) (Gandini *et al.* 2016) as well as for various hematopoietic malignancies, notably Hodgkin’s lymphomas (HL). PD1 is one of the immune response-regulating checkpoints: interaction between PD1 on T-cells and PD-L1 on cancer cells provides a mechanism for cancer cells to evade proper recognition as foreign and thus escape attack by the immune system. Currently, several monoclonal anti-PD1 or anti-PD-L1 antibodies that inhibit this interaction ‘–checkpoint inhibitors’ – are approved by the FDA for clinical use; the first was pembrolizumab, an anti-PD1 agent, initially approved for the treatment of advanced melanoma and currently also for advanced NSCLC and for recurrent or metastatic head and neck squamous cell carcinoma (http://www.accessdata.fda.gov/scripts/cder/daf/index.cfm?event=overview.process&applno=125514; accessed 17.03.2017). The second anti-PD1 agent approved was nivolumab, initially approved for the treatment of advanced melanoma and currently also for several other tumors such as advanced NSCLC; metastatic renal cell carcinoma; HL; recurrent or metastatic head and neck squamous cell carcinoma; and previously-treated locally advanced or metastatic urothelial carcinoma (http://www.accessdata.fda.gov/scripts/cder/daf/index.cfm?event=overview.process&ApplNo=125554; accessed 17.03.2017). Atezolizumab is currently the only approved anti-PD-L1 agent; it is in use for urothelial carcinoma and for metastatic NSCLC (https://www.fda.gov/drugs/informationondrugs/approveddrugs/ucm525780.htm; accessed 17.03.2017).

In several cancer types where these checkpoint inhibitors are used, clinical responses rates as high as 30% to 50% have been demonstrated (Gandini *et al.* 2016). When considering such molecular-targeted therapies for an individual patient, identification of predictive biomarkers may be useful for patient selection to improve treatment efficacy while avoiding unjustified secondary effects and also making rational use of healthcare resources. Thus, several studies have investigated the immunohistochemical expression of PD1 and PD-L1 in both tumor cells and tumor-infiltrating immune cells, showing that malignant cells are PD-L1-positive in a variable proportion of HL, melanoma, glioblastoma, NSCLC and head and neck, breast, ovarian, renal, pancreatic and esophageal carcinoma (Chowdhury *et al.* 2016).

Regarding thyroid tumors, a few papers have reported on PD-L1 expression in thyroid (Cunha *et al.* 2013, Angell *et al.* 2014, Wu *et al.* 2015, Bastman *et al.* 2016, Chowdhury *et al.* 2016, Ahn *et al.* 2017, Shi *et al.* 2017). With the exception of two studies on anaplastic (undifferentiated) thyroid carcinoma (ATC) (Wu *et al*. 2015, Ahn *et al.* 2017), these studies were focused primarily on follicular cell-derived tumors (differentiated thyroid carcinoma, DTC), including papillary thyroid carcinoma (PTC), follicular thyroid carcinoma (FTC) and poorly differentiated thyroid carcinoma (PDTC) (Cunha *et al.* 2013, Angell *et al.* 2014, Bastman *et al.* 2016, Chowdhury *et al.* 2016, Ahn *et al.* 2017, Shi *et al.* 2017). For these tumors (i.e., DTC), immunotherapy could be considered in the small minority of cases that are classed as refractory to radioiodine treatment.

**Figure 1:**
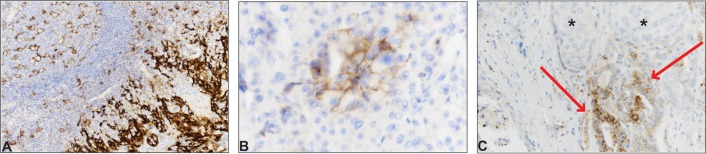
PD-L1 expression in medullary thyroid carcinoma. A. Benign tonsil tissue was used as positive control: on the right side of the picture, the reticulated crypt epithelial cells show strong membranous positivity for PD-L1; on the left side, some lymphocytes and macrophages in germinal centers show weak membranous positivity (PD-L1 immunostain, ×200). B. Focal and membranous expression for PD-L1 in malignant cells in case n° 10. The overall expression in malignant cells was scored at 5% (PD-L1 immunostain, ×400). C. One of the two cases showing PD-L1 positivity in the lymphocytic infiltrate (case n° 12); PD-L1 was expressed by reactive follicular cells (arrows) and was not expressed by malignant C-cells (asterisks) (PD-L1 immunostain, ×200).

The latest study by Ahn and coworkers recently published in *Endocrine-Related Cancer* used tissue microarrays to investigate 407 primary thyroid cancers for PD-L1 expression using the monoclonal antibody SP142 (Ahn *et al.* 2017). PD-L1 was found to be expressed in cancer cells in 6.1% of PTC, 7.6% of FTC and 22.2% of ATC; regarding immune cells, they were positive for PD-L1 in 28.5% of PTC, 9.1% of FTC and 11.1% of ATC. In general, the more aggressive the tumor, the higher the expression of PD-L1, yet no significant association was found between PD-L1 expression and disease progression, disease-free survival or other clinicopathological parameters. Interestingly, in a pilot trial of pembrolizumab in DTC, only a limited percentage of partial responses was observed (9.1%, 2/22 patients) (Mehnert *et al.* 2016). Nevertheless, other trials to test checkpoints inhibitors (https://clinicaltrials.gov/ct2/show/NCT03012620; https://clinicaltrials.gov/ct2/show/NCT02458638; accessed 17.03.2017) are ongoing for radioiodine-refractory DTC. Due to its distinct origin from parafollicular cells, medullary thyroid carcinoma (MTC) is *always* refractory to radioiodine treatment. Even though the aforementioned clinical trials will also test the effectiveness of targeting the PD1/PD-L1 system in MTC, to the best of our knowledge there are no reports on the expression of PD-L1 in MTC. We thus decided to investigate this question in all MTC patients operated in our tertiary center over a twenty-year period (1996–2016). Using an anti-PD-L1 rabbit monoclonal antibody (clone SP263, ready to use, Ventana Medical Systems, Tucson, AZ, USA), we assessed PD-L1 expression in both tumor cells and tumor-infiltrating immune cells in the tumor specimens (complete histological sections, not tissue microarray). The staining was performed with the BenchMark automated immunostainer (Ventana Medical Systems, Tucson, AZ, USA). We scored tumor cells expressing PD-L1 as a percentage of total tumor cells; we scored tumor-infiltrating immune cells expressing PD-L1 as a percentage of positive cells within the tumor area. We considered as positive only the membranous pattern, and not the cytoplasmic one, because PD-L1 is functional as a transmembrane protein. The threshold to consider the staining as positive was a percentage of stained cells >1%; for positive cases; the percentage of stained cell was recorded. As controls we used placenta and benign tonsil tissues (Fig. 1A).

Sixteen cases of MTC were recovered over the study period: 5 males and 11 females with a median age of 47 years (Table 1). All but one cases scored negatively in the tumor cells. The patient who showed positive cells (5%) was a female, and was still alive at the last follow-up; she had a microcarcinoma (0.8 cm) (Fig. 1B). Regarding tumor-infiltrating immune cells, of which there are generally few in MTC (including in this series), PD-L1 was not expressed in all cases but two, with 1% and 2% of positivity. Interestingly, the latter sample also showed reactive follicles within the MTC: these were elongated and lined by thyreocytes with abundant cytoplasm, evident nucleoli and, notably, membranous positivity for PD-L1 (Fig. 1C). No correlation was evident between PD-L1 expression and clinicopathological stage or survival in our series.

**Table 1:** Clinicopathological data and PD-L1 expression in malignant cells and immune cells.

Patient n°	Sex/age, years	**Type of initial thyroidectomy**	Size, cm	pT	pN	**PD-L1 expression, % (malignant cells)**	**PD-L1 expression, % (immune cells)**	**Status and length of follow-up (years)**
1	M/47	Total	3.2	3	1b	<1	0	Alive (1,0)
2	F/58	Total	4	2	0	0	0	Alive (1,4)
3	M/47	Hemi	2.1	2	0	<1	0	Alive (6,5)
4	F/45	Total	6.2	3	1a	1	0	Dead (0,5)
5	M/61	Total	4.5	3	1b	<1	0	Dead (6,7)
5 bis*			1.9			<1	0	
6	M/71	Total	1.5	1b	1b	1	0	Dead (1,6)
7	F/40	Total	3	3	1	0	0	Lost to follow up
8	F/64	Total	0.6	1a	0	0	0	Alive (6,5)
9	F/34	Total	2.1	3	1	0	0	Dead (9,1)
10	F/52	Hemi	0.8	1a	x	5	0	Alive (11,5)
11	F/69	Total	3.5	4a	1b	1	1	Alive (6,5)
12	F/21	Total	4.8	3	x	<1	2	Dead (5,10)
13	F/45	Hemi	8	3	x	0	0	Lost to follow up
14	F/37	Total	2.9	2	1b	0	0	Alive with MTS (8,6)
15	F/58	Total	1.3	1b	0	0	0	Alive (0,5)
16	M/32	Hemi	1.5	1b	x	0	0	Dead (13,7)

* Lymph node metastasis of patient 5.

Our results showing almost no expression of PD-L1 in MTC cells and accompanying inflammatory cells should be replicated on a larger scale in other centers. They are indicative of near uniform absence of the expression of PD-L1 in this aggressive thyroid tumor subtype. The fact that we evaluated PD-L1 expression in a large portion of each tumor’s surface (as opposed to tissue microarrays in the study by Ahn et al.) confers robustness to the present work. Another difference with the study by Ahn and coworkers is that we used a different monoclonal antibody, namely SP263; however, it has been shown that the results obtained with these two antibodies are highly correlated (Gaule *et al.* 2017).

Like DTC, MTC has one of the lowest mutational loads and neo-antigen repertoires among all solid tumors (Agrawal *et al.* 2013). Moreover, as mentioned above, on histological examination, MTC usually has very few accompanying inflammatory cells. These reasons may account for the very low expression of PD-L1 in our series. Nevertheless, experience with melanoma (a tumor more immunogenic and with more tumor-infiltrating immune cells than MTC) suggests that immunotherapy against checkpoint inhibitors can be clinically beneficial even in cases with low expression of PD-L1. Thus, definitive answers regarding the utility of PD1/PD-L1 immunophenotyping in MTC (as well as in DTC and ATC) and of the use of checkpoint inhibitors in the treatment of thyroid neoplasms must await the results of the respective ongoing clinical trials, provided that these trials will perform immunohistochemistry for PD1/PD-L1.

## Declaration of interest

The authors declare that they have no conflicts of interest that could be perceived as prejudicing the impartiality of the research reported.

## Funding

This work was partly supported by the Swiss National Science Foundation (Project 31003A_153062, 2013); the Swiss Society of Endocrinology-Diabetology (Young Independent Investigator Award, 2014); and the Leenaards Foundation (2016 Fellowship for academic promotion in clinical medicine), all to GPS.
